# Anticancer Potential of Halogen Derivatives of Methyl 6-Acetyl-5-Hydroxy-2-Methyl-1-Benzofuran-3-Carboxylate

**DOI:** 10.3390/ijms26125493

**Published:** 2025-06-08

**Authors:** Mariola Napiórkowska, Emilia Grosicka-Maciąg, Piotr Podsadni, Dagmara Otto-Ślusarczyk

**Affiliations:** 1 Chair and Department of Biochemistry, Medical University of Warsaw, 1 Banacha Str., 02-097 Warsaw, Poland; dagmara.otto@wum.edu.pl; 2 Department of Biochemistry and Laboratory Diagnostic, Collegium Medicum, Cardinal Stefan Wyszyński University, Kazimierza Wóycickiego 1 Str., 01-938 Warsaw, Poland; e.grosicka-maciag@uksw.edu.pl; 3 Department of Drug Technology and Pharmaceutical Biotechnology, Faculty of Pharmacy, Medical University of Warsaw, 1 Banacha Str., 02-097 Warsaw, Poland; piotr.podsadni@wum.edu.pl

**Keywords:** benzofurans, cytotoxicity, apoptosis, anticancer, interleukin-6, cell cycle

## Abstract

The presented results are a continuation of our research on the synthesis and biological properties of halogen benzofuran derivatives, particularly their anticancer potential. We examined the cytotoxicity of two derivatives, methyl 4-chloro-6-(dichloroacetyl)-5-hydroxy-2-methyl-1-benzofuran-3-carboxylate (**7**) and methyl 6-(dibromoacetyl)-5-methoxy-2-methyl-1-benzofuran-3-carboxylate (**8**), in the following human cancer cell lines: SW480, SW620, HCT116, HepG2, PC3, A549, and MDA. The MTT assay results showed that compound **7** exhibited the most promising activity against A549 cells, while compound **8** demonstrated significant activity against both A549 cells and HepG2 cells. The biological activity of these compounds was evaluated by the trypan blue assay, reactive oxygen species generation, lipid peroxidation and IL-6 secretion. To investigate the proapoptotic activity of these compounds, the two following types of tests were performed: Annexin V Apoptosis Detection Kit I and Caspase-Glo 3/7 assay. Moreover, we checked the effect of both tested derivatives on the cell cycle and tubulin polymerization. The obtained results revealed that the presence of bromine and methoxy group in the structure has an influence on the biological properties of compound **8**. This derivative exhibited stronger pro-oxidative effects and proapoptotic properties compared to those observed for derivative **7**. Both compounds decreased IL 6 secretion in the tested cancer cell lines; however, the stronger effect was observed for HepG2 cells. Analysis of the cell cycle in the presence of the tested compounds revealed that compound **7** induced G2/M phase arrest in HepG2 cells, while compound **8** caused cell cycle arrest at the S and G2/M phases in A549 cells. On the other hand, both derivatives had a minimal effect on tubulin polymerization. These findings suggest that compounds **7** and **8** could serve as starting points for further development of anticancer agents.

## 1. Introduction

**Figure 1:**
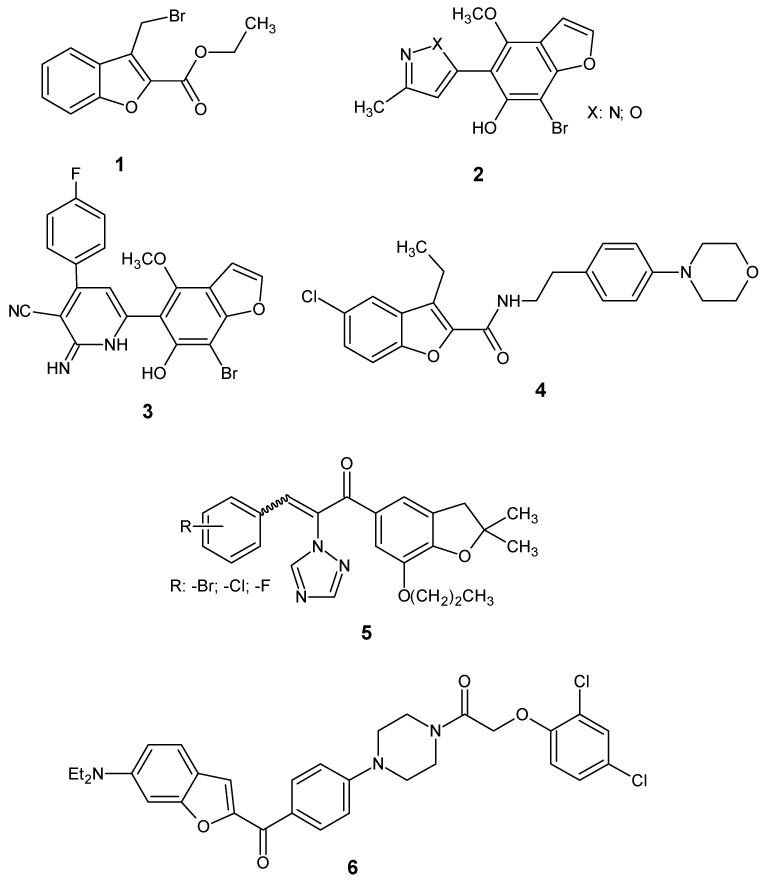
The structures of active halogen derivatives of benzofurans.

As cancer incidence continues to rise at an alarming rate, the search for new, more selective, and effective anticancer compounds has become a key focus for many researchers. Numerous research reports show that compounds based on the benzofuran skeleton exhibit multidirectional biological activity. Both natural and synthetic derivatives of this class of compounds have been shown to possess antibacterial, antifungal, antioxidant, anti-inflammatory, anticonvulsant, and anti-HIV activities [1,2,3]. Notably, many benzofuran derivatives exhibit anticancer activity without showing toxicity toward normal cells [1,2,3,4,5,6,7,8,9,10]. Among these, halogenated benzofuran derivatives represent an important class of compounds with cytotoxic activity. The introduction of halogens such as bromine, chlorine, or fluorine into the benzofuran ring has been shown to significantly enhance anticancer activity. A promising example is the ethyl ester of 3-bromomethyl-benzofuran-2-carboxylic acid (**1**, Figure 1), which was identified as a selective inhibitor of the PLK1-PBD (polo-box domain of polo-like kinase 1) and demonstrated activity against selected tumor cells both in vitro and in vivo [10]. Additionally, Amin et al. described the novel bromo-substituted benzofurans linked to various heterocyclic cores, such as N-substituted pyrazole, isoxazole, and pyridine (compounds **2** and **3**, Figure 1), which were found to act as p38α MAP kinase inhibitors and exhibit cytotoxic activity against breast cancer cells [11].

Youssif et al. reported 5-chlorobenzofuran-2-carboxamide derivatives, such as compound **4** (Figure 1), as having antiproliferative effects against tumor cells [12]. Moreover, halogenated (E,Z)-1-(dihydrobenzofuran-5-yl)-3-phenyl-2-(1,2,4-triazol-1-yl)-2-propen-1-ones with halogens substituted at the para-position of the phenyl ring (compound **5**, Figure 1) have been described as possessing antiproliferative activity with low cytotoxicity towards normal cells [13]. In another study, a series of hybrid compounds combining benzofuran and N-aryl piperazine moieties were identified as potential anticancer agents. Among them, compound **6** (Figure 1), containing chlorine in the benzene ring, was one of the most potent derivatives, showing strong activity against four human tumor cell lines: A549, HeLa, MCF-7, and SGC7901 [6].

Our team’s research has also revealed that halogen derivatives of benzofuran demonstrate cytotoxic activity against various cancer cell lines [14,15,16,17]. Notably, our results suggest that introducing halogens to alkyl or alkoxy substituents significantly increases the cytotoxicity of these compounds compared to the unsubstituted parent benzofurans. The position of the halogen appears to be a key factor in determining the cytotoxic activity of the studied benzofurans. This makes them an interesting group for further investigation as potential anticancer agents with selectivity against cancer cells over normal cells. Additionally, our studies have shown that benzofurans induce apoptosis in these cells via both receptor-mediated and mitochondrial pathways, and we have identified tubulin as the molecular target of specific compounds [16].

**Figure 2:**
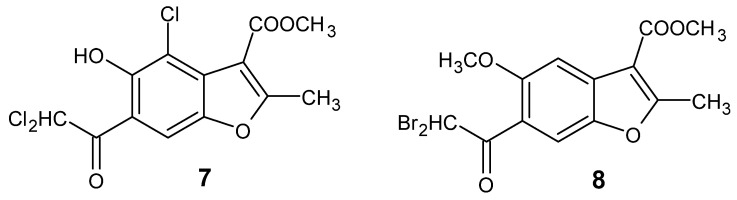
The structure of the tested compounds.

In previous investigations involving derivatives of methyl 6-acetyl-5-hydroxy-2-methyl-1-benzofuran-3-carboxylate, we identified two particularly potent compounds, labeled as **7** and **8** (Figure 2), which display significant cytotoxicity against selected cancer cell lines while showing no cytotoxicity towards HUVEC cells [15].

Based on this finding, we expanded our research on these two compounds across a panel of seven human cancer cell lines, including colon (SW480, SW620, HCT116), liver (HepG2), prostate (PC3), lung (A549), and breast cancer cells (MDA-MB-231). We examined the impact of the tested compounds on cell viability, cell cycle, apoptosis, IL-6 secretion, ROS production and lipid peroxidation. Additionally, we investigated whether compounds **7** or **8** affect tubulin polymerization in vitro.

## 2. Results and Discussion

### 2.1. Chemistry

Benzofuran derivatives **7** and **8** were synthesized following a previously documented procedure [14]. The spectra acquired (^1^H NMR, ^13^C NMR, and ESIMS) and the melting points of compounds align with prior measurements, validating the structures of the synthesized compounds. The scheme of synthesis and spectra of compounds **7** and **8** are presented in Supplementary Materials (Scheme S1, Spectra S1–S6). Purity of the compounds was established by high-performance liquid chromatography (HPLC) method and was above 95%. 

### 2.2. Biological Evaluation

#### 2.2.1. In Vitro Cytotoxic Activity

The cytotoxic activity of compounds **7** and **8** was assessed in seven human cancer cell lines using the MTT assay: prostate (PC3), colon (HCT116, SW480, SW620), liver (HepG2), lung (A549), and breast (MDA-MB-231). The results were expressed as the IC_50_—the concentration of the compound that corresponds to a 50% growth inhibition of cell lines as compared with the control. The results obtained are presented in Table 1. Doxorubicin (DOX) and cisplatin (CP) were used as reference agents. The results showed that compound **8** demonstrated stronger anticancer potential than compound **7** in all cancer cell lines, except colon cancer cells—HCT116. The most pronounced effect was observed for HepG2, A549, and SW620, with IC_50_ values of 3.8 ± 0.5 μM, 3.5 ± 0.6 μM, and 10.8 ± 0.9 μM, respectively. Compound **7** exhibited the highest cytotoxicity in A549 with an IC_50_ value of 6.3 ± 2.5 μM and, moderate in HepG2 with an IC_50_ value of 11 ± 3.2 μM. The obtained results confirm our previously presented hypothesis that the introduction of bromine into the structure increases the cytotoxic potential of benzofurans [18,19]. Additionally, brominated derivatives appear to be more cytotoxic than chlorine-containing derivatives. This may be due to differences in the electronegativity of these halogens, the size of their atomic radii, and, consequently, the polarity of the tested molecules. Furthermore, the differences in the biological activity of examined benzofurans may also impact the size and arrangement of substitutions in the benzene ring.

The correlation of cytotoxicity between the studied compounds and the reference drugs, doxorubicin, and cisplatin, revealed that both compounds were less cytotoxic than doxorubicin across all tested cell lines. However, both compounds exhibited greater cytotoxic potential in A549 cells, and compound **8** showed greater cytotoxicity in HepG2 compared to cisplatin.

Importantly, according to our previous studies, both tested derivatives did not exhibit toxic potential for normal HUVEC cells (IC_50_ > 1000µM) [15]; so, these data indicate that these compounds show high selectivity against cancer cells.

Based on the obtained results of the MTT test, we decided that the biological properties of both derivatives would be tested in two cancer cell lines for which the IC_50_ value was lower than 10 µM.

#### 2.2.2. Antiproliferative Activity

The next step of our research was an examination of the cellular viability and the total cell number after treatment with compounds **7** or **8** at an IC_50_ concentration for 72 h using the trypan blue exclusion assay.

The results presented in Table 2 demonstrate that compound **7** used at an IC_50_ concentration cause a minimal reduction in the viability of both A549 and HepG2 cells. On the other hand, it possessed antiproliferative properties and decreased significantly the total cells number of both cancer lines. Compound **7** decreased the total cell number by 66% in A549 cells and 43% in HepG2 cells. The results confirmed the higher cytotoxic potential of brominated derivative comparing to chlorinated one. Compound **8** significantly decreased cancer cell viability, concomitantly decreasing the total cell number. The most pronounced antiproliferative effect was observed for A549 cells, as the cell number decreased by almost 94% compared to the control. In the presented trypan blue study, we mainly focused on the evaluation of the antiproliferative activity of both compounds. The obtained results are in agreement with our previous research describing antiproliferative properties of benzofurans derivatives [18,19].

#### 2.2.3. ROS Production and Lipid Peroxidation Studies

Contrary to the normal cells, cancer cells demonstrate higher levels of reactive oxygen species (ROS) as a consequence of hypermetabolism, gene mutation, and relative hypoxia condition [20]. The adaptation of cancer cells to elevated ROS levels may result in increased antioxidant enzymatic and nonenzymatic mechanisms. On the other hand, excessive ROS concentration makes cancer cells more sensitive to the presence of pro-oxidants or inhibitors of the antioxidant system. Thus, anticancer therapies may rely on the ROS-inducing approach to impair cancer cells. The increase in intracellular levels of ROS in cancer cells may exert harmful effects on cell homeostasis, structures, and functions, resulting in cell death [20,21].

Literature data indicate that benzofuran derivatives can modulate oxidative stress differently in various cell types [22,23,24]. On the one hand, these compounds can act as antioxidants, preventing cells from damage, induced by excessive ROS levels. For example, novel benzofuran-2-carboxamide derivatives showed neuroprotective and antioxidant activity against NMDA (N-methyl-D-aspartic acid receptor) induced toxicity [25]. On the other hand, benzofuran derivatives may increase oxidative stress, leading to cell death, as was observed in lung cancer cell lines A549 and PC9 treated with benzofuran derivative Moracin N [26]. This compound caused ROS accumulation, consequently leading to apoptosis and autophagy of cancer cells.

**Figure 3:**
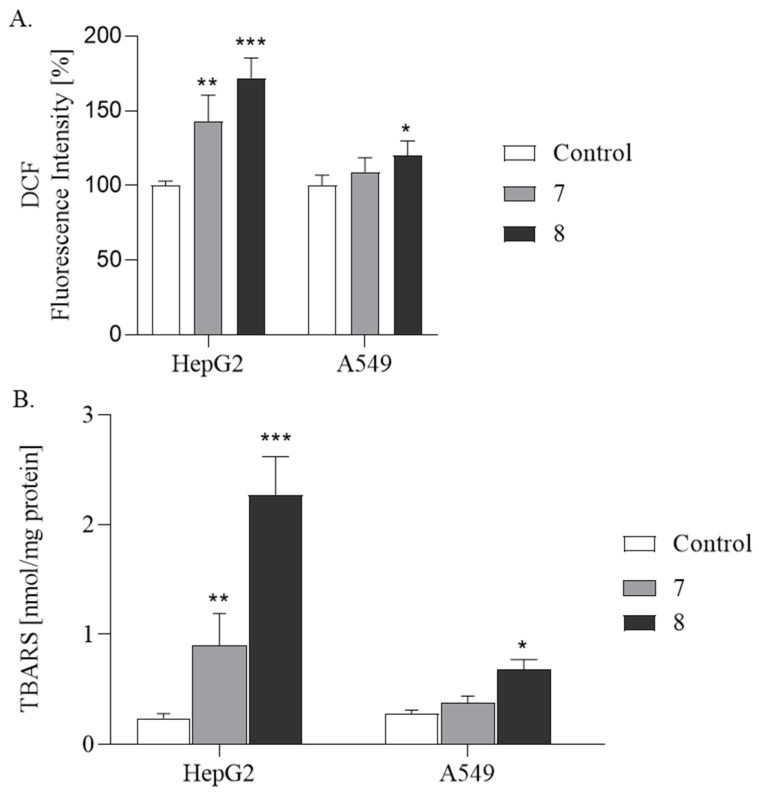
ROS production (**A**) and TBARS concentration (**B**) in HepG2 and A549 cancer cells treated with compounds **7** and **8**. The results are expressed as mean ± SD. *** *p* ≤ 0.001, ** *p* ≤ 0.01, * *p* ≤ 0.05, as compared to the control.

Based on these findings, to clarify the potential mechanism behind the cytotoxicity of compounds **7** and **8**, their impact on ROS production was examined (Figure 3A). The cells were treated with benzofurans at IC_50_ concentrations for 24 h. ROS generation was then measured spectrofluorometrically using 2′,7′-dichlorodihydrofluorescein diacetate (DCF-DA). The analysis is based on ROS-dependent oxidation of the compound to fluorescent dichlorofluorescein (DCF). The results indicated that compounds **7** and **8** exhibited pro-oxidative activity in both cell lines examined. However, a stronger effect was observed in HepG2 cells. Compound 8 increased fluorescence by 72% (*p* ≤ 0.001), whereas compound 7 increased it by 43% (*p* ≤ 0.01) compared to the control. In A549 cells, compound **8** increased fluorescence by 20% (*p* ≤ 0.05), while compound **7** resulted in a 9% not statistically significant increase. The observed increase in fluorescence means that both compounds caused elevated production of H_2_O_2_, which is responsible for oxidation of DCF-DA. To summarize, the observed changes generated by tested derivatives confirmed the thesis that benzofurans may act as a pro-oxidant. However, the pro-oxidative effect depends on the type of cancer lines and specific halogen presences in the structure. The more pronounced effect was observed in HepG2 cells. There are many studies indicating that an elevated concentration of ROS and induction of oxidative stress in HepG2 may direct cells into the apoptosis pathway and may be an important factor in anticancer therapy [27,28,29]. This experiment, one more time, shows that the bromine derivative is more biologically active than the chlorine one (compound **7**).

Our research revealed that both derivatives raised H_2_O_2_ concentrations in cells. Hydrogen peroxide is the product of the reaction catalyzed by superoxide dismutase (SOD). In this reaction, superoxide (O_2_^−^) is converted into O_2_ and H_2_O_2_. Superoxide plays roles in cell signaling and pathogen defense but can cause oxidative damage if not controlled. Hydrogen peroxide is more stable and membrane-permeable. While less reactive on its own, it becomes highly damaging through the Fenton reaction, producing hydroxyl radicals (•OH). It also participates in signaling and immune responses. Based on this fact, we can suppose that both examined compounds increased the production of O_2_^−^. Both the superoxide and the less reactive H_2_O_2_, when overproduced and not eliminated by the antioxidant systems, are responsible for peroxidation of cellular protein and lipids. We evaluated lipid peroxidation by measuring thiobarbituric reactive substances (TBARSs). The increase in TBARS production is an indicator of unsaturated fatty acid degradation by free radical attack. Uncontrolled lipid peroxidation leads to cell injury and may cause cell death. Figure 3B illustrates the influence of tested benzofurans on lipid peroxidation. The analysis of lipid peroxidation in both cells line shows once again that compound **8** exhibits a stronger pro-oxidative potential. Additionally, of the two cell lines tested, HepG2 cells show greater sensitivity to increased ROS levels. The concentration of TBARSs was higher in HepG2 cells compared to A549 cells, with a nine-fold increase for compound **8** (*p* ≤ 0.001) and a four-fold increase for compound **7** (*p* ≤ 0.01). In A549 cells, the TBARS levels were elevated 2-fold for compound **8** (*p* ≤ 0.05) and 1.5-fold for compound **7**. These results suggest that the ROS production by the tested compounds in HepG2 cells may be one of the mechanisms underlying their cytotoxic effects, leading to apoptosis or necrosis of these cells. Our results showed that halogen derivatives of benzofurans have pro-oxidant activity. However, it should be mentioned that many of the tested derivatives may have potential therapeutic effects based on their antioxidant properties, particularly in the nervous system [22,25,30,31]; on the other hand, they may act as a pro-oxidant and disrupters of antioxidant systems [18,26,32].

Summarizing this part of our work, we indicate that modifications in the structure of benzofuran derivatives significantly affect their biological properties and the effects of their action depend on the tested cell line.

#### 2.2.4. Interleukin-6 Assay

Interleukin-6 (IL-6) is an inflammatory molecule produced by various cells, including tumors and cells in the tumor microenvironment. It promotes tumorigenesis by affecting apoptosis, survival, proliferation, angiogenesis, invasiveness, metastasis, and the metabolism of cancer cells [33]. Elevated levels of IL-6 are commonly found in the serum and tumor tissues of many types of cancer. Therefore, reducing IL-6 levels or blocking its signaling pathway, STAT-3, could be a promising therapeutic approach for cancers characterized by IL-6 overproduction [33,34].

**Figure 4:**
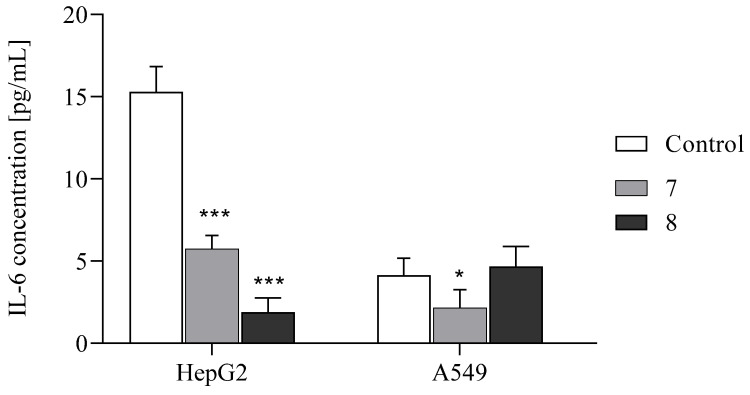
The effects of compounds **7** and **8** on IL-6 HepG2 and A549 cancer cells, measured by the ELISA test. Data are expressed as the mean ± SD. *** *p* ≤ 0.0001, * *p* ≤ 0.01, as compared to the control.

In the present study, we investigated the ability of compounds **7** and **8** to inhibit IL-6 production in cancer cell lines (Figure 4). The results showed that the highest inhibitory effect of the tested compounds on IL-6 production was observed in HepG2 cells. Compound **8** decreased IL-6 production by 87% ± 0.87 (*p* ≤ 0.0001), while compound **7** reduced it by 67% ± 0.9 compared to the control (*p* ≤ 0.0001). Interestingly, compound **8** did not affect IL-6 levels in A549 cell lines, and the effect of compound **7** was lower than in HepG2, with a 50% ± 1.1 reduction in the IL-6 level (*p* ≤ 0.05). These findings are consistent with our previous studies and suggest that the impact of the tested compounds on IL-6 production is cell-specific [18].

#### 2.2.5. Activation of Apoptosis

**Figure 5:**
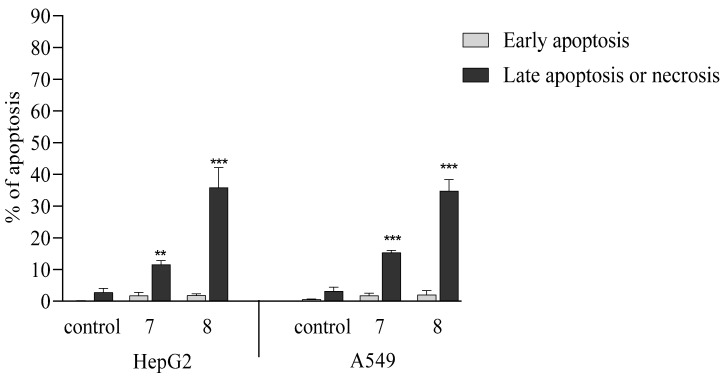
Effects of benzofuran derivatives **7** and **8** on late apoptosis or necrosis in A549 and HepG2 cells. The cells were incubated for 72 h with the respective compound at their IC_50_ concentrations; then, the cells were harvested, stained with Annexin V-FITC and PI, and analyzed using flow cytometry. Data are expressed as % of cells at the late stage of apoptosis or necrosis and as means ± SD. *** *p* ≤ 0.001, ** *p* ≤ 0.01, as compared to the control.

**Figure 6:**
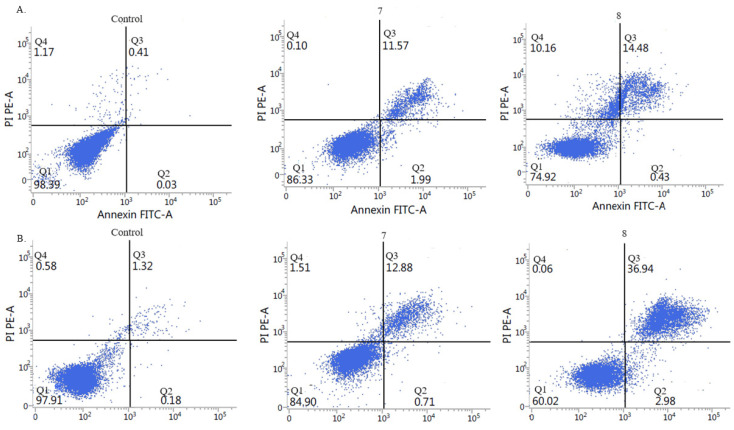
Representative results (%) as dot plots from apoptosis analysis of (**A)**. HepG2 and (**B**). A549 cancer cells treated with compounds **7** and **8**, determined by flow cytometry, using the Annexin V-FITC/PI staining bioassay.

**Figure 7:**
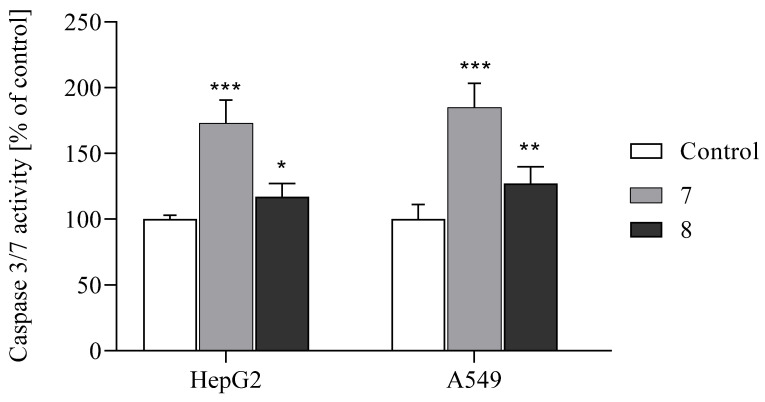
The effects of compounds **7** and **8** on caspase-3/7 activity in HepG2 and A549. The cells were incubated with the compounds at their IC_50_ concentrations for 24 h. Data are expressed as % of control and as the mean ± SD. *** *p* ≤ 0.0001, ** *p* ≤ 0.001, * *p* ≤ 0.01, as compared to the control.

To elucidate the mechanism of cytotoxicity induced by compounds **7** and **8**, we assessed the type of cell death after exposure to both derivatives. For this purpose, we used the Annexin V Apoptosis Detection Kit I (Figure 5 and Figure 6) and Caspase-Glo 3/7 assay (Figure 7), which allow for the assessment of the type of cell death induced by the tested compounds. We previously showed that both compounds increased the concentrations of H_2_O_2_ and we observed elevated lipid peroxidation after exposure to both derivatives. The accumulation of ROS may induce cellular damage and it promotes cell death. Based on this fact, we can suppose that both compounds might possess apoptotic potential. We conducted the Annexin V-FITC test, which allows us to detect the early structural changes in cellular membrane which occur during apoptosis. The obtained results showed that both compounds applied at IC_50_ concentrations induced considerably late apoptosis or necrosis in cancerous cells compared to controls. Compound 8 revealed the stronger apoptotic potential in both cell lines. The results of the flow cytometry analysis showed that it induced late apoptosis/necrosis in 36% of HepG2 cells and in 35% of A549 cells. Compound **7** also induced late apoptosis/necrosis of cancer cells but to a lesser extent. The number of apoptotic cells amounted to 15% for A549 and 12% for HepG2.

The increase in Annexin (+) cells after treatment with the tested compounds **7** and **8** suggests that executioner caspases 3/7 are involved in this process. It is well known that the presence of active caspases plays an important role in the apoptotic cell death induced by anticancer drugs. Activated by extrinsic or intrinsic apoptotic pathways, executioner caspases 3 and 7 cleave many substrates, resulting in morphological and biochemical hallmarks of apoptosis such as nuclear condensation or DNA fragmentation [35]. The activity of these caspases was measured by the Caspase-Glo 3/7 Assay in cancer cell lines exposed to benzofuran derivatives at an IC_50_ concentration for 24 h (Figure 7).

It was observed that compound **7** significantly increased the activity of caspase 3/7 in both HepG2 and A549 cells (by 73 and 85%, respectively). These results indicate that apoptosis was induced by compound **7** in a caspase-dependent way, which is consistent with our previous study conducted on benzofuran derivatives [15,16,17].

Surprisingly, although apoptotic cells were also detected in cancer cells treated with compound **8**, the activity of caspases 3/7 was lower in these cell lines compared to cells treated with compound **7**. The obtained results can be explained by the different times needed to activate caspases 3/7 depending on cell type and type of stimulant. Alternatively, derivative **8** may induce apoptosis in a caspase-independent pathway in HepG2 and A549 cells.

The obtained results align with research reports showing that benzofuran derivatives can induce apoptosis in various cancer cell lines. Furthermore, it was described that the anticancer activity of these compounds may occur through multiple mechanisms [1,4,12,32,36,37,38,39,40,41,42,43,44,45,46,47,48].

To sum up, the presented results and the results of our previous research on this group of compounds allow us to assume that these derivatives activate both receptor and mitochondrial apoptotic pathways in the cancer cell lines tested [16].

#### 2.2.6. Test Benzo [B] Furans Cause G2/M Cell Cycle Arrest

**Figure 8:**
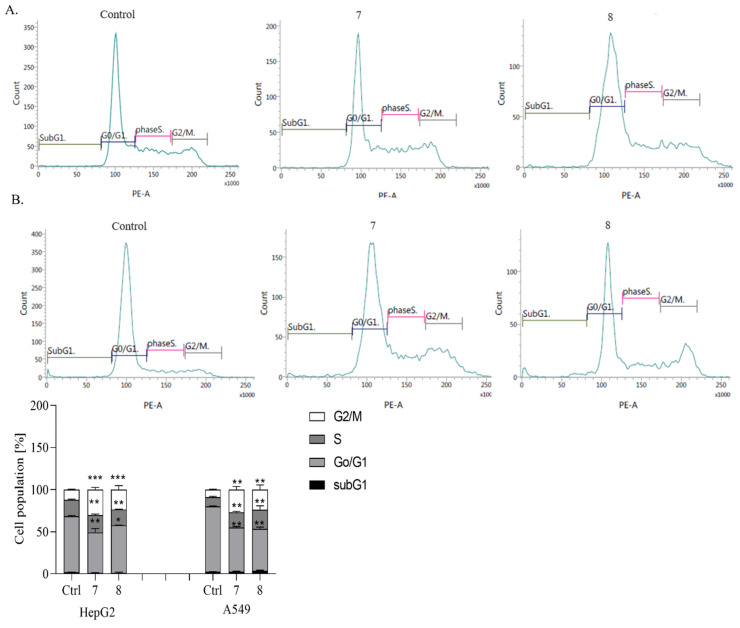
Flow cytometry analysis of cell cycle parameters after incubation of (**A**) A549 and (**B**) HepG2 cancer cells treated with compounds **7** and **8** at their IC_50_ concentrations for 24 h *** *p* < 0.001, ** *p* < 0.01, * *p* < 0.05.

Changes in the cell cycle distribution profile are known to trigger apoptosis. Therefore, in the next phase of our study, we examined, by flow cytometry analysis, the impact of both compounds used at IC_50_ concentrations on the cell cycle in the tested cancer cell lines (Figure 8).

The analysis of cell cycle distribution in HepG2 and A549 cells after 24 h exposure to both derivatives revealed a decrease in the G0/G1 cell population by approximately 20–30% and 35–40%, respectively (Figure 8).

Incubation of HepG2 cells with compounds **7** and **8** markedly increased the percentage of the cell population in the G2/M phase, from 1.35% to 10.87% (approximately eight-fold), and slightly decreased the proportion in the S phase. Meanwhile, in A549 cells, cell cycle arrest was observed predominantly in the G2/M phase, with a significant increase in the cell population by approximately 10- to 13-fold, respectively (Figure 8). Regulating cell cycle progression is crucial for maintaining homeostasis in healthy tissues and normal cells [49,50]. Therefore, inducing cell cycle arrest in cancer cells is considered an effective strategy in anticancer drug development [51,52]. Our previous studies have documented that novel derivatives of benzofurans can impede cell cycle progression, specifically arresting cells at the sub-G1 or G2/M phase depending on cancer type [16,18]. The present study similarly showed that compounds **7** and **8** induced G2/M phase arrest in HepG2 cells and G2/M arrest with a slight increase in the S phase in A549 cells. These findings collectively suggest a potential therapeutic role for benzofuran derivatives in modulating cell cycle dynamics in specific cancer types.

#### 2.2.7. Benzo [B] Furans Inhibit Tubulin Polymerization In Vitro

**Figure 9:**
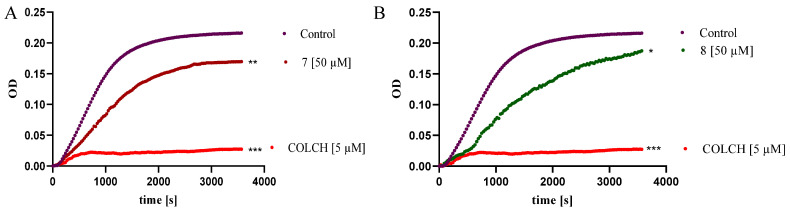
The effect of the compounds on the kinetics of in vitro tubulin polymerization. Time dependence of tubulin polymerization at 37 °C in the presence of vehicle (1% *v*/*v* DMSO, control, violet line) or the compounds 7 (**A**) and 8 (**B**) at 50 µM concentration as indicated, measured by turbidometry at 340 nm. The results were compared using one-way ANOVA analysis with Dunnett’s post hoc in comparison to the control *** *p* < 0.0001, ** *p*< 0.001, * *p*< 0.001.

Research reports indicate that the molecular target of some benzofuran derivatives with anticancer activity is microtubules [46,47]. Microtubules, as a key component of the cytoskeleton, play an important role in the cell’s life functions: they ensure the integrity of its structure and determine its shape, are responsible for intracellular transport, acting as “communication pathways”, participate in cell movement as components of cilia and flagella, and are involved in morphogenetic processes (e.g., in the formation of axons and spermatozoa). Microtubules are primarily the main component of the mitotic spindle, responsible for the segregation of chromosomes into daughter cells during mitosis and meiosis. Composed of alpha/beta heterodimers, these are not static but highly dynamic polymers, and maintaining the proper balance between their polymerization and depolymerization is crucial for their functions in the cell. Disruption of this balance can lead to cell cycle arrest, mitotic blockage, inhibition of cell proliferation, and ultimately cell death [48]. Due to these functions, microtubules have become a significant molecular target in the development of anticancer drugs [53]. The results of our previous studies showed that tubulins are targets for the halogenated benzofurans, which inhibited tubulins’ polymerization in a manner comparable to that of the reference drug vinblastine [16]. Thus, we decided to investigate whether the antiproliferative effects of compounds **7** and **8** are due to their action on the tubulin–microtubule system. The impact of compounds **7** and **8** on microtubule dynamics was analyzed using a Tubulin Polymerization Assay Kit. Colchicine (COLCH), a well-known microtubule-destabilizing agent, was used as a reference compound. The kinetics of microtubule formation were monitored by measuring absorbance at 340 nm over time. Figure 9 shows the relative degree of tubulin polymerization in the presence of the tested compounds. The highest optical density (OD) of the control sample (containing 1% DMSO) was arbitrarily set to represent 100% tubulin polymerization. In the presence of COLCH (5 μM), tubulin polymerization was almost completely inhibited, resulting in only 9% polymerized tubulin. In contrast, compounds **7** (Figure 9A) and **8** (Figure 9B) (50 μM) were weaker inhibitors, resulting in 72% and 82% polymerized tubulin, respectively. The observed weak inhibitory effect on tubulin polymerization indicates that tubulins are not targets for examined derivatives. Based on this, we suppose that antiproliferative effects and apoptotic activity of both compounds might result from mechanisms which occur on the other molecular pathway.

## 3. Materials and Methods 

### 3.1. Chemistry

The tested benzofurans **7** and **8** were synthesized according to the procedure reported previously [14] and a brief description and a characterization of the structures are presented below (Scheme S1).

All commercial reagents, solvents and chemicals used in the present studies were purchased from Aldrich Chemical Company (Saint Louis, MO, USA) and Alfa Aesar (Haverhill, MA, USA) and used without purification.

The melting points of the compounds were determined in open capillaries in an Electrothermal 9100 apparatus (Elektrothermal, Kraków, Poland). Nuclear magnetic resonance (NMR) spectra were recorded on a Bruker BioSpin GmbH spectrometer (Bruker, Billerica, MA, USA) operating at 300 MHz (^1^H NMR) and 75.49 MHz (^13^C NMR) in DMSO-d_6_ or CDCl_3_. Chemical shifts (δ) are expressed in parts per million relative to tetramethylsilane (TMS), used as the internal reference. Coupling constants (*J*) values are given in hertz (Hz), and spin multiples are given as s (singlet), d (dublet), t (triplet), m (multiplet). Mass spectra (MS) measurements were carried out on a Quadrupole Time-of-Flight Liquid Chromatograph Mass Spectrometer (LCMS-9030, Shimadzu, Kyoto, Japan). The spectrum was obtained in a negative ion mode for compound **7** and a positive ion mode for compound **8**. The compounds were purified by column chromatography using silica gel (Merck, Rahway, NJ, USA, Kieselgel 0.05–0.2 mm, 70–325 mesh ASTM). The reactions were monitored by thin-layer chromatography (TLC) on silica gel plates (fluorescent indicator at 254 nm, layer thickness 0.2 mm, Kieselgel G, Merck) with chloroform as the eluent.

Purity of the compounds was established by high-performance liquid chromatography (HPLC) method and was above 95.5% (Supplementary Tables S1–S4). Shimadzu NEXERA UHPLC System consisting of binary pumps (LC-30AD), autosampler (SIL-30AC), column oven (CTO-20AC), diode array detector (SPD-M20A), and controller CBM-20A was used, and Gemini-NX C18 150 × 3 mm column (Phenomenex, Torrance, CA, USA) with corresponding precolumn. Temperature of column oven was set at 35 °C and injection volume was 10 µL. The analytical wavelength was 320 nm.

#### 3.1.1. Synthesis of Methyl 4-Chloro-6-(Dichloroacetyl)-5-Hydroxy-2-Methyl-1-Benzofuran-3-Carboxylate (**7**, Figure 2)

Methyl 6-acetyl-5-hydroxy-2-methyl-1-benzofuran-3-carboxylate was dissolved in 20 mL of chloroform (CHCl_3_). Chlorine gas, generated in situ by the reaction of potassium permanganate (KMnO_4_) with concentrated hydrochloric acid (HCl), was then passed through the solution. Upon completion of the reaction, the solvent was evaporated under reduced pressure. The resulting residue was purified by column chromatography on silica gel, using chloroform and a chloroform/methanol (100:0.5) mixture as eluents.

Yield: 36%; m.p. 171–172 °C; ^1^H-NMR δ (ppm): 11.70 (s, 1H, OH), 7.98 (s, 1H, C7-H), 6.73 (s, 1H, COCHCl_2_), 3.96 (s, 3H, COOCH_3_), 2.68 (s, 3H, CH_3_); ESI MS m/z: 350.8:352.6 [M+H]+ (100%); ^13^C NMR (75 MHz, CDCl_3_), δ (ppm): 190.40, 167.98 (2xC,C(O)); 163.25, 156.32, 146.18, 132.75, 113.82, 111.02, 110.92 and 110.16 (8xC, C aromatic); 67.46 (1xC, Cl-CH-Cl); 52.09 (1xC, -OCH3); 14.58 (1xC, -CH3); HRMS (ESI) calc. for C_13_H_9_Cl_3_O_5_ [M-H]^−^: 349.95156; m/z 348.94428, found: 348.94350 (Spectra S1–S3).

#### 3.1.2. Synthesis of Methyl 6-(Dibromoacetyl)-5-Methoxy-2-Methyl-1-Benzofuran-3-Carboxylate (**8**, Figure 2)

Methyl 6-acetyl-5-methoxy-2-methyl-1-benzofuran-3-carboxylate (0.02 mol) was dissolved in 20 mL of chloroform (CHCl_3_). A solution of bromine (0.04 mol) in 10 mL of CHCl_3_ was then added dropwise over 30 min with continuous stirring. The reaction mixture was stirred for an additional 8 h at room temperature. After the reaction was complete, the solvent was evaporated under reduced pressure. The resulting residue was purified by column chromatography on silica gel using chloroform as the eluent.

Yield: 78%; m.p. 135–138 °C; ^1^H-NMR δ (ppm): 7.92 (s, 1H, C7-H), 7.52 (s, 1H, C4-H), 7.20 (s, 1H, COCHBr_2_), 4.03 (s, 3H, COOCH_3_), 3.96 (s, 3H, OCH_3_), 2.78 (s, 3H, CH_3_); ^13^C NMR (75 MHz, CDCl_3_), δ (ppm): 187.52, 167.89 (2xC,C(O)); 164.12, 155.74, 148.14, 132.62, 119.13, 114.41, 109.17 and 103.46 (8xC, C aromatic); 56.45 (1xC, Br-CH-Br); 51.67 (1xC, -OCH3), 44.70 (1xC, -OCH3); 14.98 (1xC, -CH3). HRMS (ESI) calc. for C_14_H_12_Br_2_O_5_ [M+H]^+^: 417.90515; m/z 418.91243, found: 418.91175 (Spectra S4–S6).

### 3.2. Biological Studies

#### 3.2.1. Human Cell Line, MTT Assay

The studies were conducted according to the methods described earlier [18,19]. The experimental procedure summary for these studies is presented in Table 3.

#### 3.2.2. Measurement of Live Cell Number and Viability (%) by Trypan Blue Exclusion Assay

This study was conducted according to the methods described earlier [18,19]. The experimental procedure summary for this study is presented in the table below (Table 4).

#### 3.2.3. Measurement of Cell Apoptosis by Flow Cytometry Annexin V-FITC Binding Assay

To assess apoptosis, the cells were stained with Annexin V-FITC and propidium iodide (PI) using a commercial apoptosis detection kit (BD Biosciences Pharmingen, San Diego, CA, USA), following the manufacturer’s instructions. The cells (5 × 10^4^/well) were seeded in 12-well plates. After 24 h, the cells were treated with compounds **7** or **8** at an IC_50_ concentration. Untreated cells were included as negative controls. After 72 h of incubation, both non-adherent and adherent cells were harvested for analysis. Dual staining with Annexin V-FITC and PI enabled the identification of early apoptotic (Annexin V^+^/PI^−^) and late apoptotic or necrotic (Annexin V^+^/PI^+^ or Annexin V^−^/PI^+^) populations. Samples were analyzed using flow cytometry (Becton Dickinson, San Jose, CA, USA). Each experiment was conducted independently three times to ensure reproducibility, and the results are expressed as the mean ± standard deviation.

#### 3.2.4. Cell Cycle Analysis

The cells (1 × 10^5^ per well) were plated in six-well plates and incubated overnight to allow for adherence. The next day, they were exposed to the test compounds at their respective IC_50_ concentrations and incubated for an additional 24 h. Following treatment, both adherent and non-adherent cells were harvested and centrifuged at 400 × *g* for 5 min at 4 °C. The collected cells were washed twice with phosphate-buffered saline (PBS) and fixed in 500 μL of cold 70% ethanol at 4 °C overnight.

Before analysis, the cells were centrifuged again at 850 × *g* for 5 min at 4 °C and washed with PBS. They were then incubated with 50 μL of RNase (100 µg/mL; Sigma-Aldrich) and stained with 200 μL of propidium iodide (PI; 50 μg/mL; EMD Millipore, Billerica, MA, USA) at 37 °C for 30 min in the dark. After staining, 100 μL of PBS was added to each sample. The distribution of cells across different phases of the cell cycle—sub-G1, G0/G1, S, and G2/M—was assessed using flow cytometry (Becton Dickinson).

#### 3.2.5. Caspases-3 and -7 Activity Assay

Caspase-3/7 activity was assessed using the Homogeneous Caspase-Glo^®^ 3/7 Assay Kit (Promega, Madison, WI, USA), following the manufacturer’s protocol. Briefly, the cells (1 × 10^4^ per well) were seeded into white-walled 96-well plates and incubated at 37 °C with 5% CO_2_ for 24 h to allow for adherence. After incubation, the cells were treated with the test compounds at their respective IC_50_ concentrations. The Caspase-Glo 3/7 Reagent was brought to room temperature, and 100 µL was added to each well. The plates were then incubated at room temperature for 2 h. Following this, luminescence was measured using a Synergy HTX Multi-Mode Reader (BioTek Instruments, Charlotte, VO, USA). Background luminescence was determined using a blank containing culture medium with DMSO (the vehicle for the test compounds), while untreated cells were included as a negative control.

#### 3.2.6. DCFH-DA Assay

Reactive oxygen species (ROS) production was assessed using a spectrofluorometric technique employing 2′,7′-dichlorodihydrofluorescein diacetate (DCFH-DA) as the indicator. This method is based on the conversion of DCFH-DA into fluorescent dichlorofluorescein (DCF) by ROS [54], and a brief description of the method is shown Table 5.

#### 3.2.7. TBA Assay

Lipid peroxidation products were quantified using the thiobarbituric acid (TBA) assay, originally described by Buege and Aust in 1978, and modified by Seibert and colleagues [55]. This assay is designed to detect malondialdehyde (MDA), a key product of lipid oxidation, and other substances that react with TBA to form a colored complex. The experimental protocol for compounds **7** and **8** is presented in Table 6.

#### 3.2.8. Interleukin-6

The concentration of IL-6 in the culture medium was determined using an ELISA kit (Diaclone SAS, Besançon Cedex, France), following the manufacturer’s instructions. The cells (1 × 10^5^ /well) were seeded in 12-well plates and incubated for 24 hours. Next, the cells were treated with the test compounds at their respective IC_50_ concentrations and incubated for 72 h. After treatment, the culture medium was collected for analysis.

Finally, absorbance was measured at 450 nm using a spectrophotometer MultiscanGo spectrophotometer (ThermoFisher Scientific, Carlsbad, CA, USA). This experimental procedure was repeated on three separate occasions.

#### 3.2.9. Tubulin Polymerization

The polymerization of purified bovine tubulin was evaluated using a BK004P kit from Cytoskeleton Inc. (Denver, CO, USA), following the manufacturer’s guidelines and standard assay conditions. In summary, porcine tubulin protein (>97% purity, 3 mg/mL) was incubated at 37 °C in a buffer consisting of 80 mM PIPES (pH 6.9), 0.5 mM EGTA, 2.0 mM MgCl2, 1 mM GTP, and 5% glycerol. This mixture was prepared in the presence of either a vehicle control (1% (*v*/*v*) DMSO), colchicine (5 µM), or the tested compounds **7** and **8** at concentrations of 50 µM. Light scattering, proportional to the concentration of polymerized microtubules, was measured to monitor tubulin assembly. The polymerization process was tracked by turbidometry at 340 nm using a MultiscanGo spectrophotometer (ThermoFisher Scientific, Carlsbad, CA, USA) for 60 min. The results, showing the time dependence of tubulin polymerization, were presented as graphs created using GraphPad Prism 8.4.3.686 (Boston, MA, USA) software.

#### 3.2.10. Statistical Analysis

Statistical analysis was conducted using GraphPad Prism 9 software (GraphPad Software). Data were presented as mean ± SD from at least three independent experiments. Differences between values were evaluated using analysis of variance (ANOVA) followed by Dunnett’s multiple comparison post hoc test. A *p*-value of less than 0.05 was considered statistically significant.

## 4. Conclusions

In the presented work, we have analyzed two compounds, **7** and **8**, which exhibit selective cytotoxic potential against HepG2 and A549. The evaluation of biological properties revealed that the brominated compound seems to be more active than the chlorinated one; moreover, it demonstrated a stronger potential in HepG2 cells. The most pronounced biological activity of compound **8** may also be a result of the presence of a metoxy group (-OCH_3_) in the structure, which may influence the polarity character of derivative 8 and its bioavailability. The mechanism of their biological cellular activity is based on ROS generation and oxidative stress induction expressed as lipid peroxidation. As a consequence of disturbance of the cellular antioxidant system, we observed cell death on the late apoptosis/necrosis pathway. Additionally, compound **8** significantly decreased IL6 secretion in HepG2 cells; in A549 cells, the effect was not so expressive. We also showed that both compounds modulated the cell cycle, but, on the other hand, they exhibited very weak potential as inhibitors of tubulin polymerization. Our research team has been conducting research on the synthesis and biological potential of benzofurans derivatives for many years. Over the years, we have designed, synthesized and characterized many new halogenated benzofurans derivatives, and some of them have demonstrated interesting biological and therapeutic potential. In our reports, we described derivatives with antifungal, bacterial and cancer activities. The results of our studies indicate that both the nature of the halogen atom and its position in the benzofuran ring have an impact on the biological properties of the tested benzofurans. The present research complements the knowledge about this group of halogenated benzofurans and may be used as a valuable base for further, more specific molecular studies.

## Figures and Tables

**Table 1:** Cytotoxic activity (IC_50_, µM) of studied compounds estimated by the MTT assay ^a^.

Compound		Cancer Cells					Normal Cells	
IC_50,_ µM ^b^								
	SW480 ^c^	SW620 ^d^	HCT116 ^e^	HepG2 ^f^	PC3 ^g^	A549 ^h^	MDA ^i^	HUVEC ^j^
**7**	33.2 ± 1.2	26.5 ± 1.8	36.9 ± 4.1	11 ± 3.2	43 ± 2.1	6.3 ± 2.5	29.5 ± 1.5	>1000 [15]
**8**	27.6 ± 1.1	10.8 ± 0.9	58.3 ± 7.9	3.8 ± 0.5	33.2 ± 1.1	3.5 ± 0.6	23.2 ± 2.7	>1000 [15]
**Dx ^k^**	0.75 ± 0.1	0.26 ± 0.1	0.6 ± 0.02	0.4 ± 0.1	0.59 ± 0.1	0.63 ± 0.2	0.83 ± 0.03	1.0 ± 0.03
**Cp ^k^**	10.4 ± 0.9	6.70 ± 1.1	0.6 ± 0.02	4.5 ± 1.2	13.2 ± 2.1	7.10 ± 1.3	3.95 ± 1.1	12.0 ± 1.8

^a^ Data are expressed as mean SD, ^b^ IC_50_ (µM)—the concentration of the compound that corresponds to a 50% growth inhibition of cell line (as compared to the control) after culturing the cells for 72 h with the individual compound. ^c^ Human primary colon cancer (SW480), ^d^ human metastatic colon cancer (SW620), ^e^ human colon cancer (HTC-116), ^f^ human liver cancer (HepG2), ^g^ human metastatic prostate cancer (PC3), ^h^ human lung cancer (A549), ^i^ human breast cancer (MDA-MB-231), ^j^ human umbilical vein endothelial cells (HUVEC). ^k^ The selected reference compounds commonly used in cancer treatment (doxorubicin, cisplatin).

**Table 2:** The effect of tested compounds on the live cell number and viability (%) in HepG2 and A549 measured by the trypan blue assay.

Cancer Cell Line	Compound	Cell Number × 10^6^	Cell Number (%)	Viability (%)
**HepG2**	**-**	1.2 ± 0.45	100	94 ± 1.52
	**7**	0.6 ± 0.02 **	56.8	92 ± 4.93
	**8**	0.47 ± 0.01 ***	39.4	55 ± 1.10
**A549**	**-**	1.8 ± 0.90	100	97 ± 2.52
	**7**	0.6 ± 0.10 **	33.9	91 ± 1.65
	**8**	0.1 ± 0.02 ***	6.21	85 ± 7.10

The cells were incubated for 72 h with the tested compound at IC_50_ concentrations. Then, the cells were stained with trypan blue, and analyzed using a cell counter. Data are expressed as the mean ± SD. *** *p* < 0.0001, ** *p* < 0.001 as compared to control. - “control without compound”.

**Table 3:** MTT assay. Experimental procedure summary.

Step	Description
Cell Lines Used	SW480 (primary colon cancer), SW620 (lymph node colon cancer), HCT116 (colon carcinoma), PC3 (metastatic prostate cancer), HepG2 (liver cancer), A549 (lung cancer), MDA-MB-231 (breast cancer).
Cell Source	All cell lines were obtained from ATCC (Manassas, VA, USA).
Culture Media	- SW480, SW620, HCT116: MEM (ThermoFisher Scientific, Waltham, MA, USA). - PC3: RPMI. - A549, MDA-MB-231, HepG2: DMEM High Glucose (Biowest SAS, Nuaillé, France).
Media Supplements	10% FBS (Sigma-Aldrich, St. Louis, MO, USA), 20 mM HEPES (Biowest, Nuaillé, France), 100 U/mL penicillin, 100 μg/mL streptomycin (Gibco, Grand Island, NY, USA).
Culture Conditions	37 °C, 5% CO_2_, humidified incubator; cells used at 80–90% confluence.
Seeding for Assay	1 × 10^4^ cells/well in 96-well plates; adhered for 24 h.
Treatment	Cells treated with compounds **7** and **8** at various concentrations (1–100 μM) for 72 h.
Cytotoxicity Assay	MTT assay (0.5 mg/mL), incubation for 4 h.
Detection	Formazan dissolved in DMSO/isopropanol (1:1, *v*/*v*); absorbance measured at 570 nm using a MultiscanGo spectrophotometer (ThermoFisher, Waltham, MA, USA).
Data Analysis	% cytotoxicity = [A]/[B] × 100, where [A] = treated absorbance, [B] = control absorbance [54]. IC_50_ calculated using GraphPad Prism 8.0.1.

**Table 4:** Trypan blue exclusion assay. Experimental procedure summary.

Step	Description
Cell Seeding	1 × 10^5^ cells per well were seeded in 12-well plates.
Incubation	Cells were cultured for 24 h at 37 °C with 5% CO_2_ to allow for adherence.
Treatment	Cells were treated with compounds **7** or **8** at their respective IC_50_ concentrations. Untreated cells served as controls.
Incubation Post Treatment	Cells were incubated for 72 h.
Washing	Medium was removed; cells were washed twice with PBS.
Cell Harvesting	Cells were trypsinized.
Viability and Count Assessment	Cell number and viability were assessed using the trypan blue exclusion assay and an automated cell counter (Countess, Invitrogen).
Reproducibility	All experiments were performed in triplicate.

**Table 5:** Analysis of reactive oxygen species (ROS) production using DCFH-DA assay.

Step	Description
Cell Seeding	1 × 10^4^ cells/well in black 96-well plates.
Initial Incubation	24 h at 37 °C with 5% CO_2_ for cell attachment.
Treatment	Cells treated with test compounds at their IC_50_ concentrations; incubated for an additional 24 h under the same conditions.
Probe Incubation	Cells washed with PBS, then incubated with 5 μM DCFH-DA for 30 min at 37 °C in the dark.
Controls	- Positive control: 1.5 mM H_2_O_2_. - Negative control: untreated cells with no added reagents.
Fluorescence Measurement	Measured using a Microplate Spectrofluorometer (BioTek Synergy, BioTek Instruments, Winooski, VT, USA): - Excitation: 492 nm. - Emission: 527 nm.
Output	ROS level was quantified in terms of fluorescence intensity (FI).

**Table 6:** Lipid peroxidation analysis via thiobarbituric acid reactive substance (TBARS) assay.

Step	Description
Cell Lines Used	HepG2 and A549.
Cell Seeding	5 × 10^4^ cells/well in 12-well plates.
Initial Incubation	24 h at 37 °C, 5% CO_2_ to allow for cell attachment.
Treatment	Cells treated with test compounds at IC_50_ concentrations for 48 h. Untreated cells served as control.
TBARS Measurement	Post treatment, TBARS levels assessed by reading absorbance at 532 nm using a Thermo Scientific MultiscanGo spectrophotometer.
Quantification	TBARS expressed as nanomoles of MDA equivalents per milligram of protein, using the molar extinction coefficient of 1.56 × 10^5^ M^−1^ × cm^−1^.
Protein Determination	Protein content measured by the Bradford assay (separate cultures), with absorbance read at 595 nm (MultiscanGo, ThermoFisher Scientific, Carlsbad, CA, USA).

## Data Availability

Data will be made available on request.

## Supplementary Materials

The following supporting information can be downloaded at: https://www.mdpi.com/article/10.3390/ijms26125493/s1.
