# Supplementary material for: Anticancer Potential of Halogen Derivatives of Methyl 6-Acetyl-5-Hydroxy-2-Methyl-1-Benzofuran-3-Carboxylate
Source: Int J Mol Sci. 2025 Jun 8;26(12):5493. doi: 10.3390/ijms26125493 (PMC12192851; doi:10.3390/ijms26125493)
Supplement: Supplementary file 1 [file ijms-26-05493-s001.zip › ijms-3596194-supplementary.pdf]

Supplementary information

Anticancer Potential of Halogen Derivatives of Methyl 6-Acetyl-5-Hydroxy-2-Methyl-1-Benzofuran-3-Carboxylate

Mariola Napiórkowska [1,*], Emilia Grosicka-Maciąg [2], Piotr Podsadni [3] and Dagmara Otto-Ślusarczyk [1]

[1] Chair and Department of Biochemistry, Medical University of Warsaw, 1 Banacha Str., 02-097 Warsaw, Poland; dagmara.otto@wum.edu.pl

[2] Department of Biochemistry and Laboratory Diagnostic, Collegium Medicum, Cardinal Stefan Wyszyński University, Kazimierza Wóycickiego 1 Str., 01-938 Warsaw, Poland; e.grosicka-maciag@uksw.edu.pl
[3] Department of Drug Technology and Pharmaceutical Biotechnology, Faculty of Pharmacy, Medical University of Warsaw, 1 Banacha Str., 02-097 Warsaw, Poland; piotr.podsadni@wum.edu.pl

* Correspondence: mariola.napiorkowska@wum.edu.pl; Tel.: +48-22-572-06-93

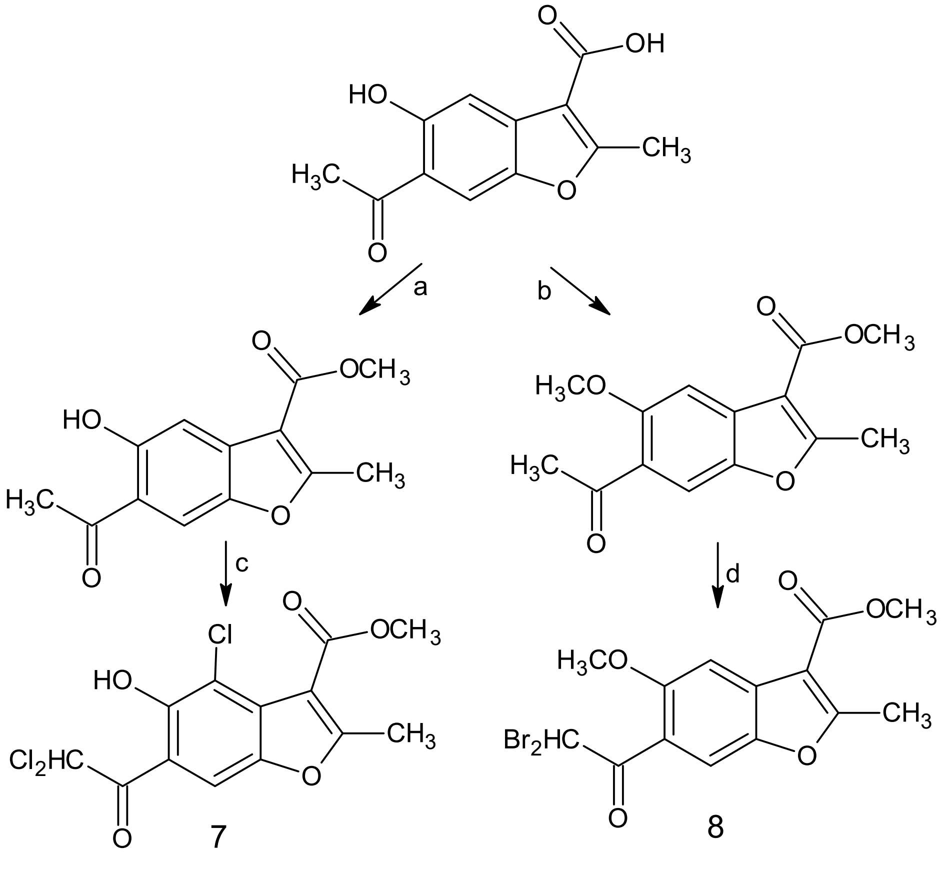

Conditions of reactions: (a) – $(CH_3O)_2SO_2$, K_2CO_3, acetone, refluxed, 5h; (b) – excess of $(CH_3O)_2SO_2$, K_2CO_3, acetone, refluxed, 10h; (c) – Cl_2 generated in reaction of $KMnO_4$ and HCl, stirred at room temperature; (d) -Br_2, CH_3Cl, stirred at room temperature, 9h

Scheme **S1**. Synthesis of the tested compounds **7** and **8**.

Spectrum S1. ^{1}HNMR of **compound 7** (300 MHz, $CDCl_3$).

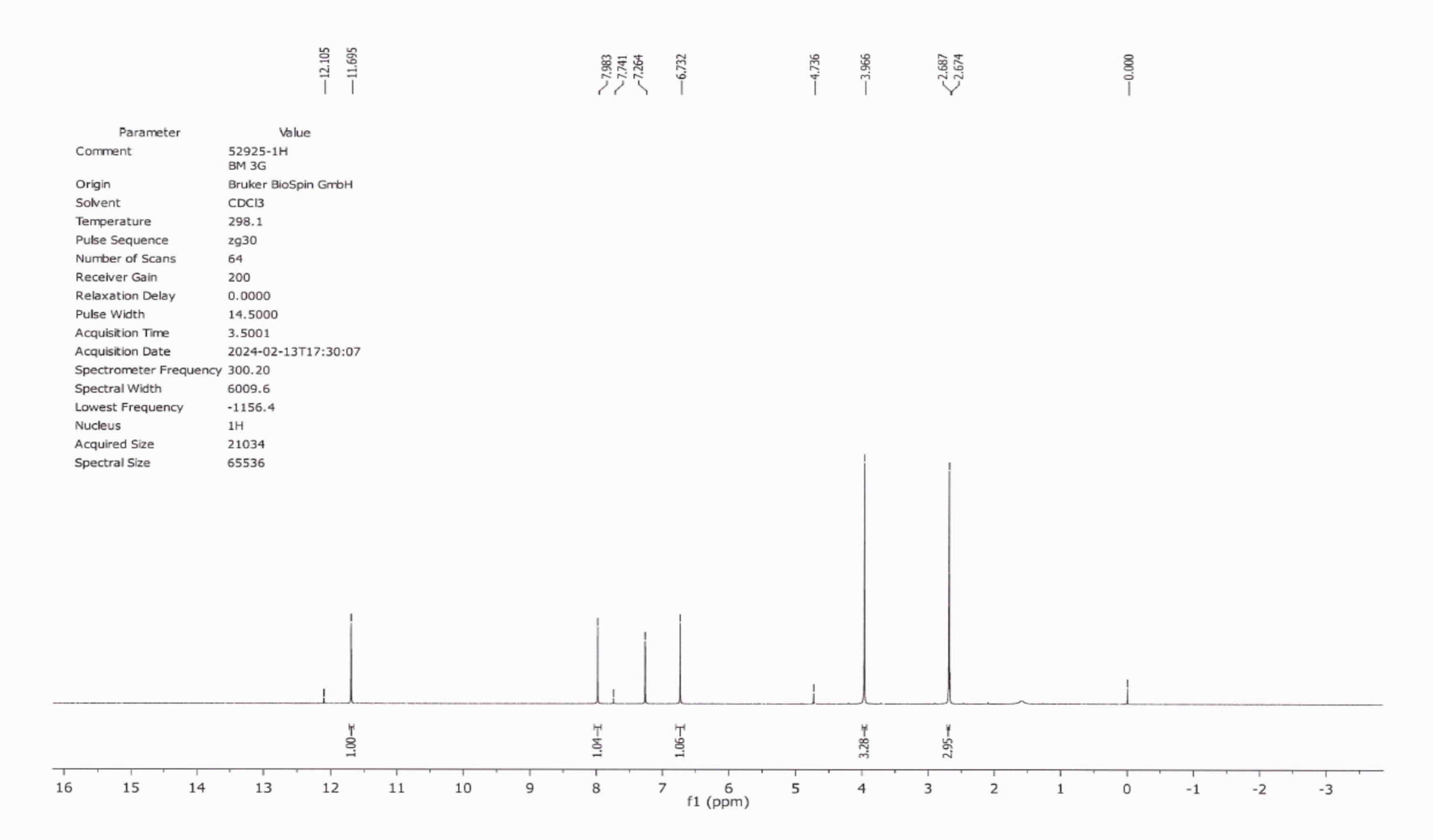

Spectrum S2. ^{13}CNMR of **compound 7** (75.5 MHz, $CDCl_3$).

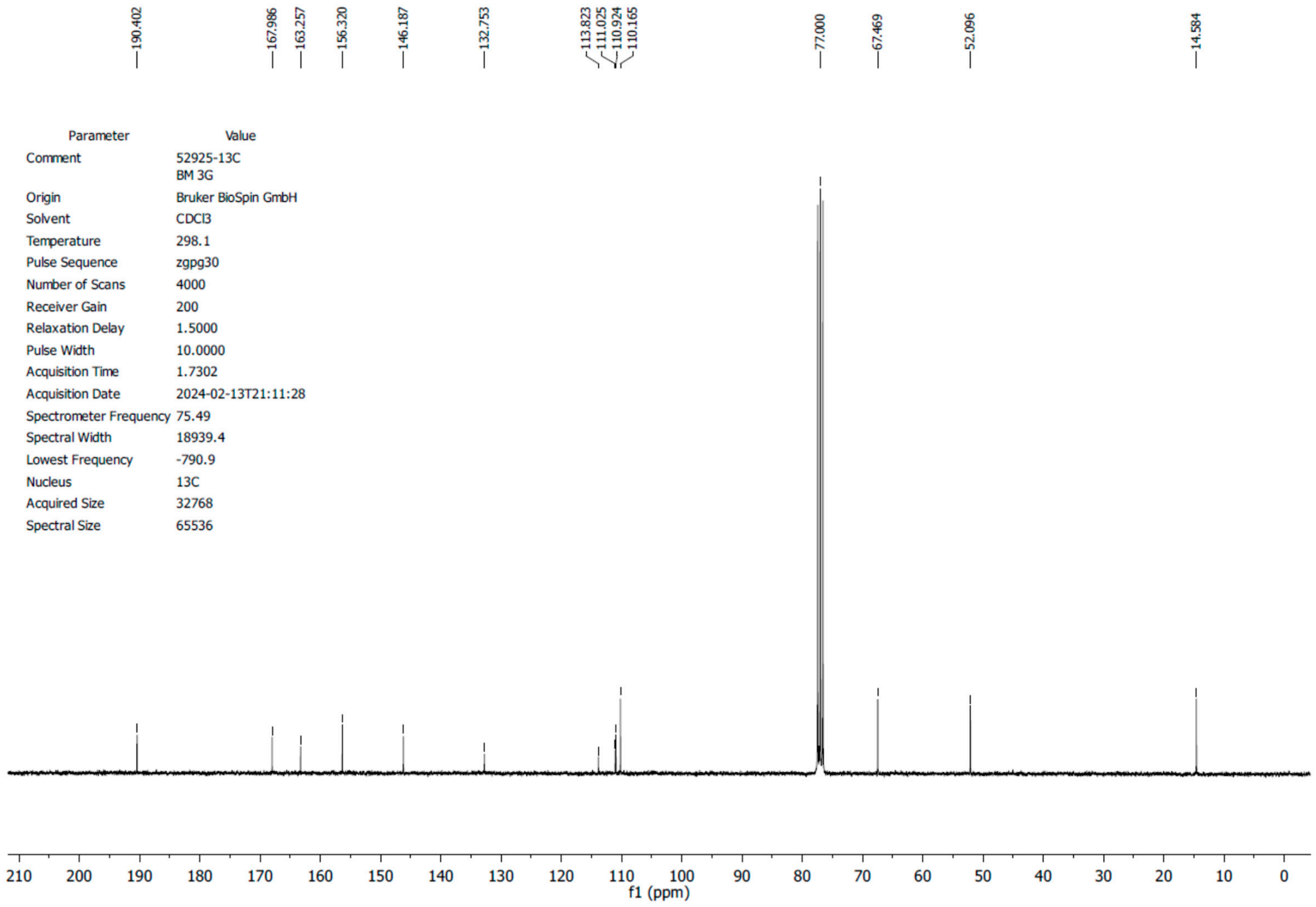

Parameter	Value
Comment	52925-13C BM 3G
Origin	Bruker BioSpin GmbH
Solvent	CDCl3
Temperature	298.1
Pulse Sequence	zgpg30
Number of Scans	4000
Receiver Gain	200
Relaxation Delay	1.5000
Pulse Width	10.0000
Acquisition Time	1.7302
Acquisition Date	2024-02-13T21:11:28
Spectrometer Frequency	75.49
Spectral Width	18939.4
Lowest Frequency	-790.9
Nucleus	13C
Acquired Size	32768
Spectral Size	65536

Spectrum S3. HRMS of **compound 7**

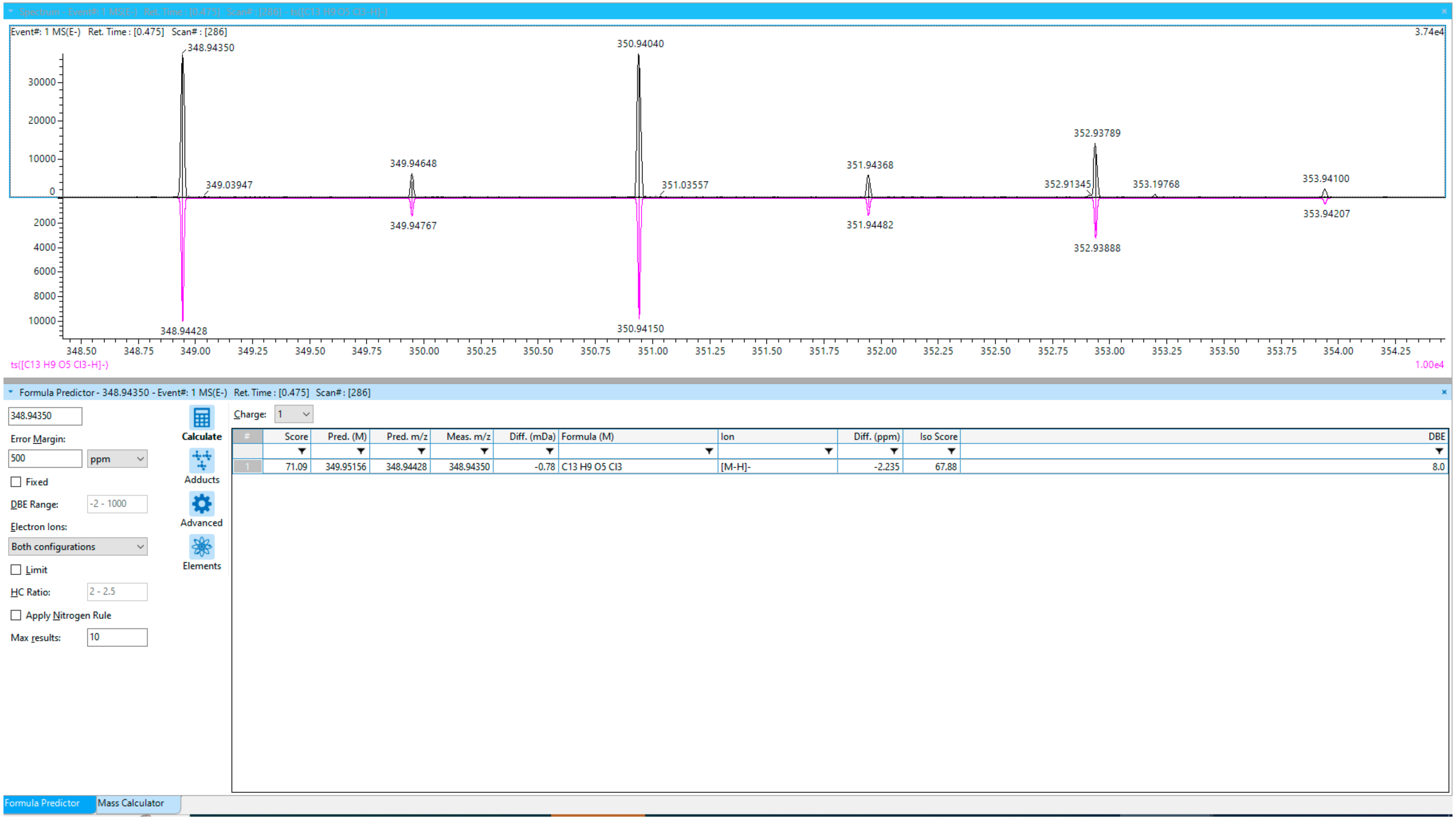

Spectrum S4. ^{1}HNMR of **compound 8** (300 MHz, $CDCl_3$).

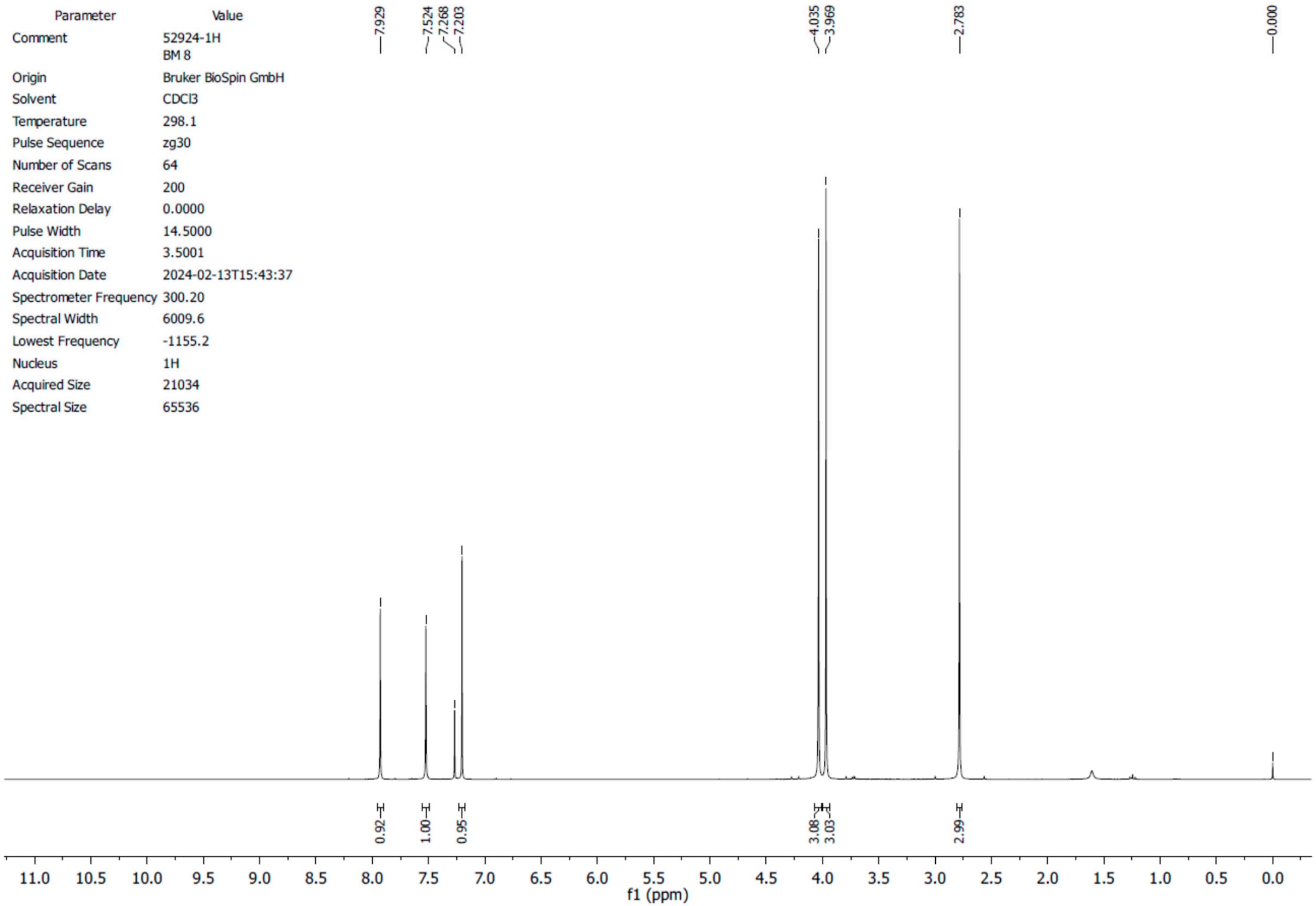

Spectrum S5. ^{13}CNMR of **compound 8** (75.5 MHz, $CDCl_3$).

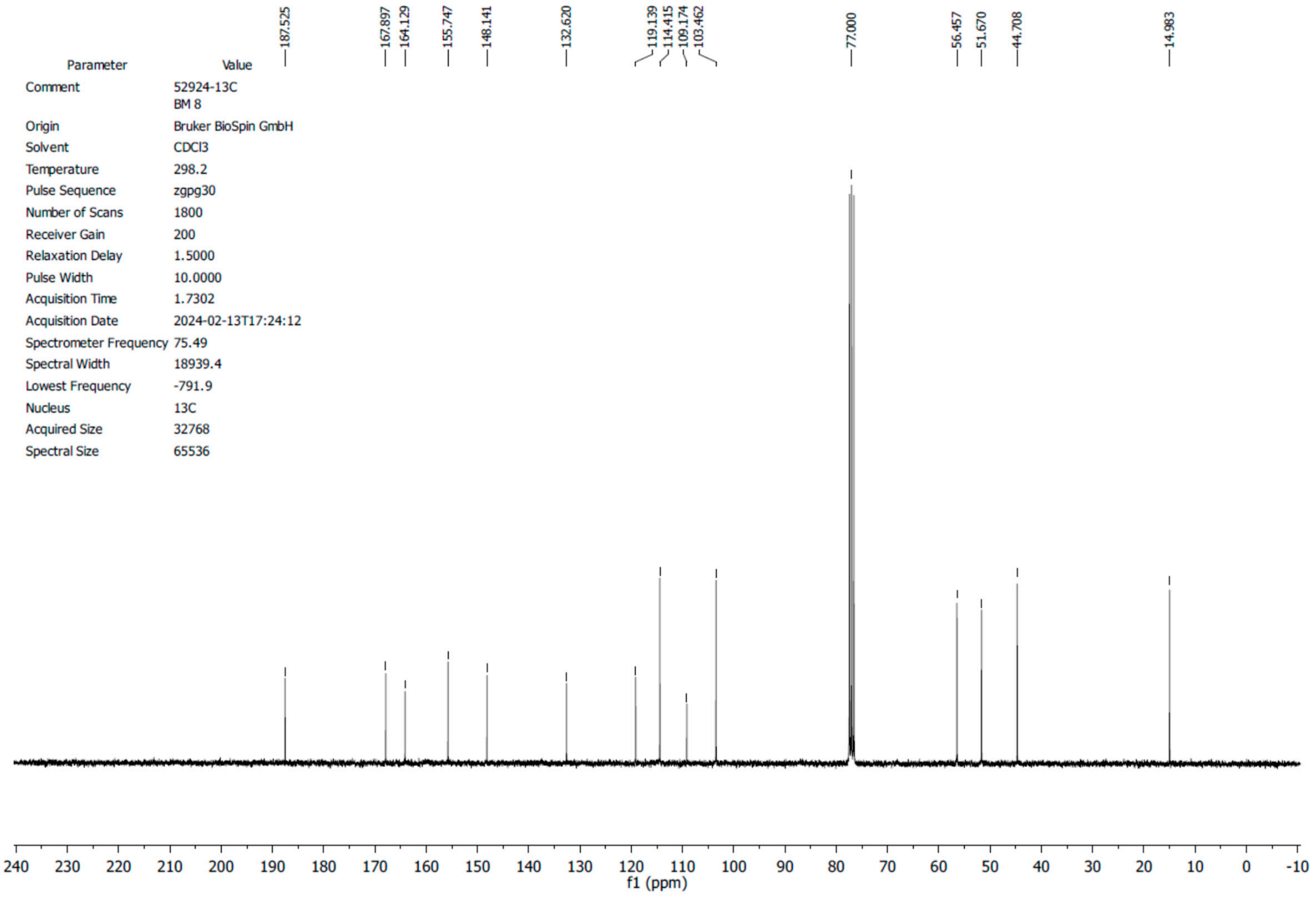

Spectrum S6. HRMS of **compound 8**

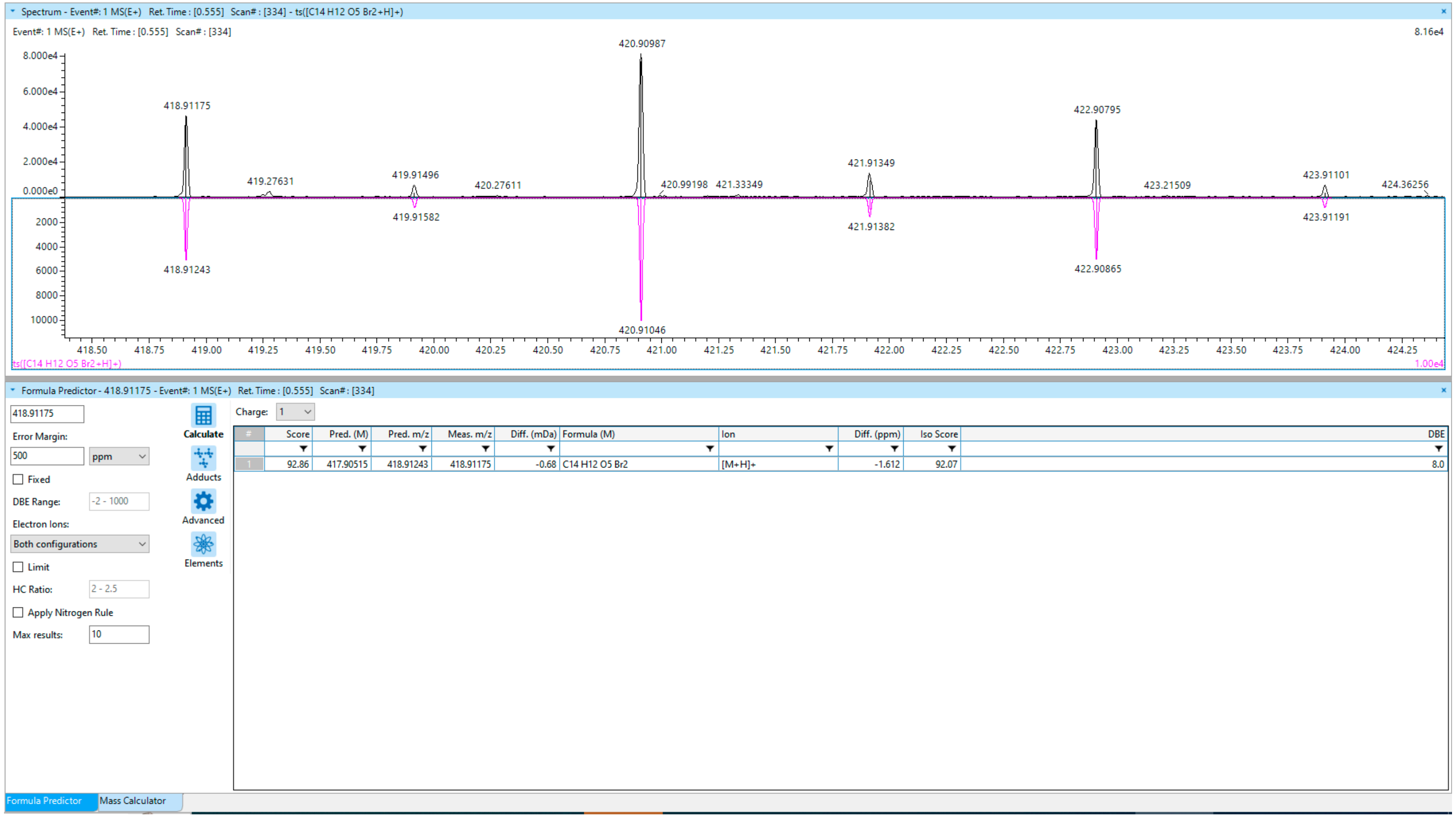

#	Score	Pred. (M)	Pred. m/z	Meas. m/z	Diff. (mDa)	Formula (M)	Ion	Diff. (ppm)	Iso Score	DBE
1	92.86	417.90515	418.91243	418.91175	-0.68	C14 H12 O5 Br2	[M+H]+	-1.612	92.07	8.0

Determination of purity of compounds 7 and 8

Sample preparation

Stock solution was prepared by dissolving 5mg of each substances in methanol (HPLC grade - POCh) in 10ml volumetric flasks. Then solutions were diluted ten times in such manner, that final solutions had 50 to 50 ratio of water to methanol. Solutions were filtered using PTFE syringe filter (0,22um, 13mm) before analysis.

Method development and chromatographic equipment and condition

To ensure that most of impurities are detected two methods of separation were used. For both methods Shimadzu NEXERA UHPLC System (Japan) was employed. It consisted of two pumps (LC-30AD), column oven (CTO-20AC), autosampler (SIL-30AC), diode array detector (SPD-M20A) and controller CBM-20A and Gemini-NX C18 150 x 3 mm column (Phenomenex, USA) with corresponding precolumn. Column oven temperature was set at 35 deg. C and injection volume was 10ul. All analysis of impurities were done at 320nm.

First method separation relied on methanol (HPLC grade -POCh) as eluting agent and second method – on acetonitrile (HPLC grade -Merck). Water phase (HPLC grade -POCh) was mixed with trifluoracetic acid (VWR) which helped to achieve good peak shapes.

Method 1. Phase A: 0,1% (V/V) TFA in water, phase B: Methanol. Total flow was set at 0,2ml/min and gradient: from 0 to 5,0 min phase B was maintained at 70%, from 5,0 to 9,0 min phase B was increased from 70% to 90% and from 9,0 to 11,5min phase B was maintained at 90%, then after 11,5 min column was reequilibrated at 70% phase B.

Method 2. Phase A: 0,1% (V/V) TFA in water, phase B: Acetonitrile. Total flow was set at 0,2ml/min and gradient was as follows: from 0 to 0,1 min phase B was maintained 50%, from 0,1 to 6,0 min phase B was increased from 50% to 80% and from 6,0 to 7,5min phase B was maintained at 80%, then after 7,5min column was reequilibrated at 50% phase B.

Test 1		Test 2	
ret. time [min]	purity [%]	ret. time [min]	purity [%]
7,264	95,821	6,908	95,361

Table S1. Purity of compound **7**

Test 1				Test 2			
**Peak#**	Ret. Time	Area%	Height%	**Peak#**	Ret. Time	Area%	Height%
**1**	2,756	0,065	0,084	**1**	3,040	0,024	0,019
**2**	3,109	0,024	0,038	**2**	3,325	0,054	0,046
**3**	4,058	0,066	0,089	**3**	5,143	0,096	0,100
**4**	5,137	3,885	6,002	**4**	5,990	3,656	3,899
**5**	7,264	95,821	93,524	**5**	6,356	0,634	0,656

**6**	9,227	0,022	0,048	**6**	6,908	95,361	95,107
**7**	11,054	0,102	0,160	**7**	7,734	0,029	0,031
**8**	11,599	0,016	0,055	**8**	8,102	0,014	0,018
**Total**		100,000	100,000	**9**	8,250	0,055	0,056
				**10**	8,663	0,029	0,022
				**11**	8,954	0,046	0,047
				**Total**		100,000	100,000

Table S2 Compound **7** and its impurity

**Test 1**		**Test 2**	
ret. time [min]	purity [%]	ret. time [min]	purity [%]
9,281	96,668	7,733	96,887

Table S3. Purity of compound **8**

**Test 1**				**Test 2**			
**Peak#**	Ret. Time	Area%	Height%	**Peak#**	Ret. Time	Area%	Height%
**1**	2,200	0,19	0,179	**1**	2,496	0,084	0,074
**2**	2,766	0,285	0,365	**2**	3,077	0,276	0,219
**3**	2,928	0,220	0,250	**3**	3,425	0,163	0,132
**4**	3,195	0,199	0,194	**4**	3,793	0,223	0,178
**5**	4,090	1,668	1,729	**5**	4,911	1,455	1,249
**6**	6,635	0,099	0,092	**6**	5,360	0,127	0,131
**7**	6,880	0,209	0,170	**7**	6,654	0,300	0,222
**8**	9,281	96,660	96,416	**8**	7,420	0,022	0,026
**9**	10,018	0,381	0,485	**9**	7,733	96,887	97,273
**10**	10,625	0,080	0,118	**10**	8,222	0,366	0,397
**Total**		100,000	100,000	**11**	8,653	0,098	0,098
				**Total**		100,000	100,000

Table S4 Compound **8** and its impurity
